# ZNF746/PARIS overexpression induces cellular senescence through FoxO1/p21 axis activation in myoblasts

**DOI:** 10.1038/s41419-020-2552-7

**Published:** 2020-05-12

**Authors:** Ju-Hyeon Bae, Hyeon-Ju Jeong, Hyebeen Kim, Young-Eun Leem, Dongryeol Ryu, Sang Chul Park, Yun-Il Lee, Sung Chun Cho, Jong-Sun Kang

**Affiliations:** 1 Department of Molecular Cell Biology, Sungkyunkwan University School of Medicine, Suwon, 440-746 Republic of Korea; 2 Single Cell Network Research Center, Sungkyunkwan University School of Medicine, Suwon, 440-746 Republic of Korea; 3 Well Aging Research Center, Daegu Gyeongbuk Institute of Science and Technology (DGIST), Daegu, 42988 Republic of Korea

**Keywords:** Senescence, Diagnostic markers

## Abstract

Various stresses, including oxidative stress, impair the proliferative capacity of muscle stem cells leading to declined muscle regeneration related to aging or muscle diseases. ZNF746 (PARIS) is originally identified as a substrate of E3 ligase Parkin and its accumulation is associated with Parkinson’s disease. In this study, we investigated the role of PARIS in myoblast function. PARIS is expressed in myoblasts and decreased during differentiation. PARIS overexpression decreased both proliferation and differentiation of myoblasts without inducing cell death, whereas PARIS depletion enhanced myoblast differentiation. Interestingly, high levels of PARIS in myoblasts or fibroblasts induced cellular senescence with alterations in gene expression associated with p53 signaling, inflammation, and response to oxidative stress. PARIS overexpression in myoblasts starkly enhanced oxidative stress and the treatment of an antioxidant Trolox attenuated the impaired proliferation caused by PARIS overexpression. FoxO1 and p53 proteins are elevated in PARIS-overexpressing cells leading to p21 induction and the depletion of FoxO1 or p53 reduced p21 levels induced by PARIS overexpression. Furthermore, both PARIS and FoxO1 were recruited to p21 promoter region and Trolox treatment attenuated FoxO1 recruitment. Taken together, PARIS upregulation causes oxidative stress-related FoxO1 and p53 activation leading to p21 induction and cellular senescence of myoblasts.

## Introduction

Skeletal muscle is maintained by stem cells called satellite cells, and upon injury quiescent satellite cells are activated, proliferated, and differentiated to regenerate muscles^1–3^. Various pathological conditions are linked with the decline of muscle mass and functionality^3^. The impaired regenerative capacity associated with muscle aging is linked with the reduced number and function of satellite cells due to failure to retain quiescent state and increased senescence^4–6^. Oxidative stress is thought to be a main factor for cellular senescence driven by the accumulation of DNA damage and irreversible cell cycle arrest to accelerate stem cell aging and the progression of degenerative diseases^5,7^. Senescent cells exhibit various characteristics such as large flat morphology, senescence-associated β-galactosidase (SA-β-Gal) activity and upregulation of cyclin-dependent kinase inhibitors p16^Ink4a^ (p16) and p21^Waf1/Cip1^ (p21) leading to cell cycle arrest^4,5,8^. Despite the well-characterized senescent phenotype markers, molecular regulatory pathways for satellite cell senescence are largely unknown. Deregulation of cell cycle inhibitors appears to be the critical mechanisms underlying senescence^4,7,8^.

Among diverse downstream targets of oxidative stress, FoxO transcription factors are recently proposed to play key roles in induction of cellular senescence^9–11^. FoxO transcription factors are originally identified as the downstream of insulin/insulin growth factor signaling, which is repressed by protein kinase B (PKB/AKT) through^12,13^. Interestingly, forced expression of FoxO1 can induce apoptosis in certain cancer cell types, whereas in other cancer cell types, it can induce G1 cell cycle arrest through upregulation of the cyclin-dependent kinase inhibitor p27^12,14^. Moreover, in response to the stimulation of transforming growth factor-β, FoxO proteins form a complex with activated Smad proteins to induce the expression of p21, leading to cell cycle arrest^15,16^. Thus, the diverse functions of FoxO proteins are dependent on the cellular contexts likely linked with distinct regulatory proteins.

ZNF746 (PARIS) has been originally identified as a substrate of E3 ligase Parkin and its accumulation has been observed in brains of Parkinson’s patients^17^. Although the molecular mechanism of PARIS is not completely understood, PARIS suppresses the transcription of peroxisome proliferator-activated receptor-γ coactivator 1-α (PGC-1α), the master regulator of mitochondria biogenesis, through binding to the insulin response element (IRE), the same consensus sequence for *FoxOs* in the *PGC-1α* promoter region^17,18^. In addition, PARIS is implicated in regulation of invasion and epithelial to mesenchymal transition of lung cancer cells and in promotion of colorectal cancer progression via enhancing c-Myc stability^19^. However, the detailed molecular mechanisms and other targets of PARIS need to be characterized. In this study, we explored the role of PARIS in the control of myoblast function. Forced expression of PARIS in myoblasts suppresses myogenic differentiation, whereas PARIS depletion enhances differentiation. PARIS overexpression elicits reduced proliferation and cellular senescence with p21 upregulation. Consistently, the transcriptome analysis of PARIS overexpression reveals dysregulation of genes related to cytokine signaling and cell cycle inhibition. PARIS overexpression triggers oxidative stress and impaired myoblast proliferation, which is rescued by Trolox treatment. Here we demonstrate FoxO1 and p53 are as targets of PARIS-induced oxidative stress leading to p21 expression and cellular senescence. Collectively, our results provide evidence that PARIS is a critical regulator to promote myoblast senescence likely contributing to impaired muscle regeneration.

## Results

### PARIS overexpression attenuates myoblast differentiation

**Fig. 1:**
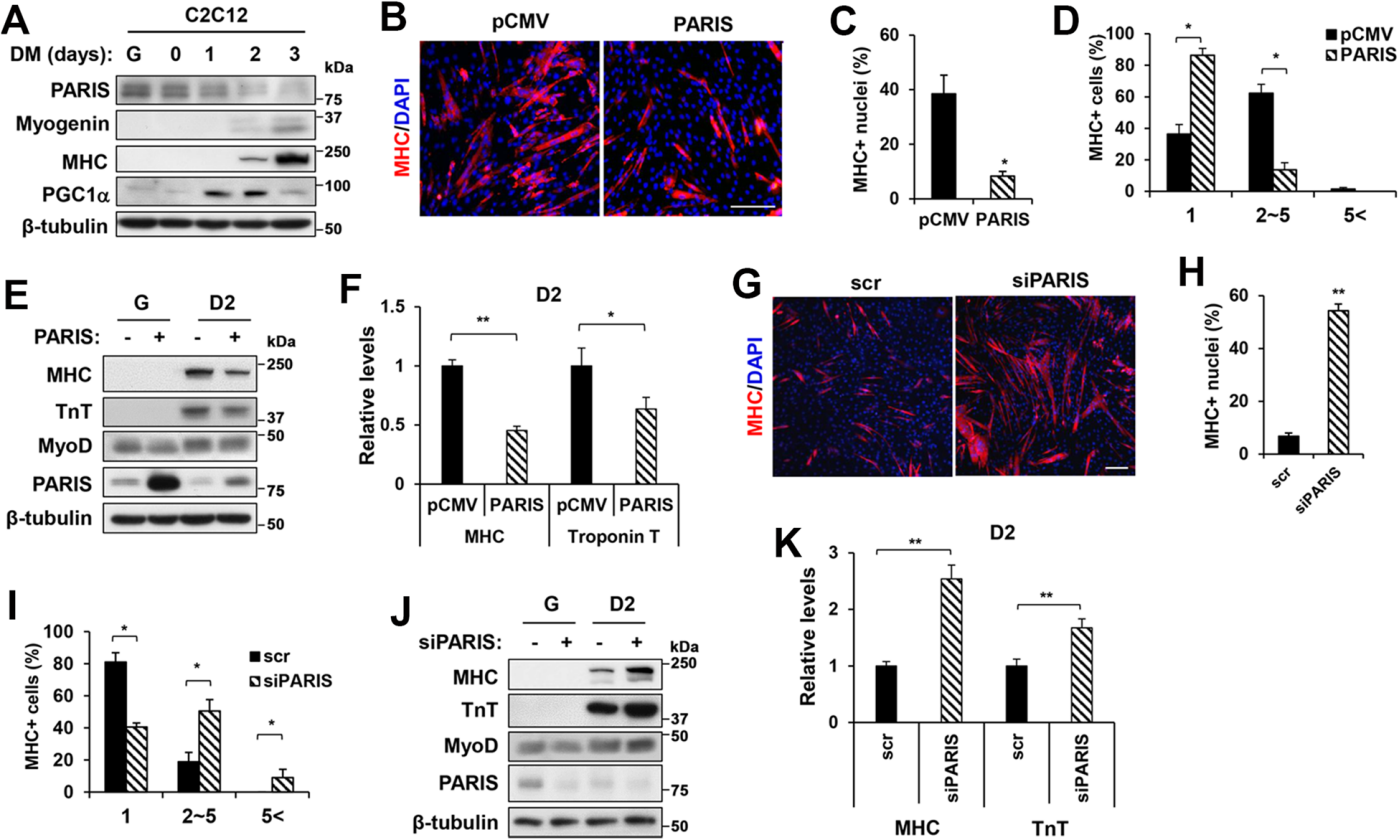
PARIS inhibited myogenic differentiation. **a** The expression of PARIS, Myogenin, MHC and PGC-1α was analyzed by immunoblotting. β-Tubulin serves as a loading control. **b** Immunofluorescence staining of MHC (red) in pCMV- or pCMV-PARIS-expressing C2C12 cells. Nuclei were visualized by DAPI (blue). Scale bar = 100 μm. **c**, **d** The percentage of nuclei and myotubes containing indicated myonuclei number was determined (*n* = 3 per group, 356 ~ 411 (pCMV) and 377 ~ 401 (PARIS) cells per sample were counted, respectively). **e** The expression of MHC, TnT, MyoD, and PARIS proteins in pCMV- or pCMV-PARIS-overexpressing C2C12 cells was analyzed by immunoblotting. **f** Quantification of the relative protein levels (three sets per group). **g** Immunofluorescence staining of MHC (red) in Scrambled (Scr)- or PARIS siRNA (siPARIS)-expressing C2C12 cells. Scale bar = 100 μm. **h**, **i** Quantification of the percentage of nuclei and myotubes containing indicated myonuclei (*n* = 3 per group, 635 ~ 1017 (pCMV) and 789 ~ 924 (PARIS) cells per sample were counted, respectively). **j** The expression of MHC, TnT, MyoD, and PARIS was analyzed by immunoblotting with Scr- or siPARIS-expressing C2C12 cells. **k** Quantification of the relative protein levels of MHC and TnT (three sets per group). Significant difference was determined by Student *t*-test (*p < 0.05, **p < 0.01).

To examine the role of PARIS in myoblast function, the expression of PARIS was examined during C2C12 myoblast differentiation. The expression of PARIS was gradually reduced during myoblast differentiation, whereas the level of PGC-1α was elevated in myoblast differentiation (Fig. 1a and Supplementary Fig. 1a). Next, control pCMV- or PARIS-overexpressing C2C12 cells were differentiated for 3 days (D3), followed by immunostaining for myosin heavy chain (MHC). C2C12/PARIS cells formed predominantly mononucleated MHC-positive myocytes and only a small proportion of myotubes contained two to five nuclei, whereas C2C12/pCMV cells formed larger myotubes (Fig. 1b–d). Consistently, the protein expression of myogenic markers, MHC and Troponin T (TnT) was significantly decreased in C2C12/PARIS cells, relative to control (Fig. 1e, f). To deplete PARIS, two different small interference RNAs (siRNAs) were tested and siPARIS-1 was used in a further study (Supplementary Fig. 1b). PARIS depletion greatly enhanced myotube formation at D2 compared with the scrambled siRNA-expressing cells (Fig. 1g–i). Moreover, the protein level of MHC and TnT was elevated in PARIS-depleted cells compared with the control scrambled siRNA-expressing cells (Fig. 1j, k). Taken together, PARIS inhibits myogenic differentiation.

### PARIS overexpression reduced myoblast proliferation

**Fig. 2:**
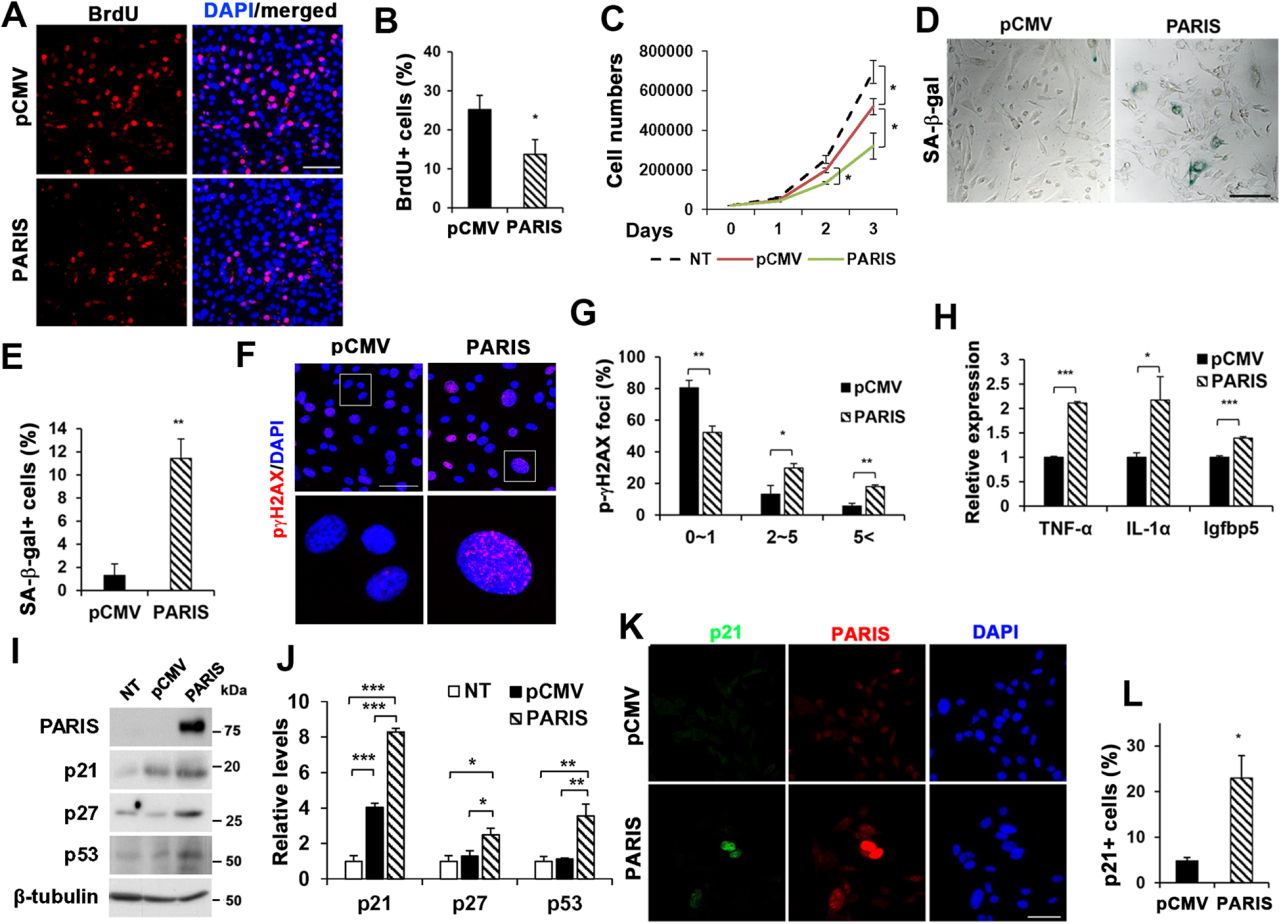
PARIS reduced myoblast proliferation while elicited cellular senescence. **a** BrdU incorporation assay was performed with pCMV- or pCMV-PARIS-overexpressing C2C12 cells. Nuclei were visualized by DAPI staining. Scale bar = 100 μm. **b** The percentage of the number of BrdU-positive cells (*n* = 4 per each groups, 349 ~ 374 (pCMV) and 244 ~ 378 cells per sample were counted). **c** Cell proliferation was determined by viable cell counting analysis using trypan blue in non-transfected (NT)-, pCMV-, or pCMV-PARIS-overexpressing C2C12 cells. Initial cell number was 2 × 10^4^ (0 day). **d** SA-β-Gal staining in pCMV- or pCMV-PARIS-overexpressing C2C12 cells at G-phase. **e** Quantification of SA-β-gal-positive cells (*n* = 3 per group, 74 ~ 84 (pCMV) and 71 ~ 81 (pCMV) cells per field were counted, respectively). **f** pCMV- or pCMV-PARIS-overexpressing C2C12 cells were immunostained against phospho-γH2AX (red). Scale bar = 50 μm. **g** Quantification of p-γH2AX foci numbers (*n* = 3 per group, 117 ~ 150 (pCMV) and 53 ~ 67 (PARIS) cells per field were counted, respectively). **h** Real-time qRT-PCR analysis for mRNA expression levels of *TNF-α*, *IL-1α*, and *Igfbp5* in pCMV- or pCMV-PARIS-overexpressing C2C12 cells. These values were normalized to *L32* (three sets per group). **i** Immunoblotting for PARIS, p21, p27, and p53 was performed in NT-, pCMV-, or pCMV-PARIS-overexpressing C2C12 cell. **j** The relative protein expression levels were quantified (three sets per group). **k** Immunostaining of p21 (green) and PARIS (red) in pCMV- or pCMV-PARIS-overexpressing C2C12 cells. Scale bar = 50 μm. **l** Quantification of p21-positive cells (*n* = 4 per group, 160 ~ 184 (pCMV) and 103 ~ 210 (PARIS) cells per field were counted, respectively). Significant difference was determined by Student *t*-test (**b**, **e**, **g**, **l**) and ANOVA (**c**, **j**) (*p < 0.05, **p < 0.01, ***p < 0.001).

As the proliferation and differentiation of myoblasts are mutually exclusive, we next asked whether PARIS inhibits myoblast differentiation through the promotion of proliferation by 5-bromo-2′-deoxyuridine (BrdU) incorporation assay. PARIS overexpression in myoblasts caused a significant reduction in the proportion of BrdU-positive cells compared with control (Fig. 2a, b). Furthermore, cell counting experiments revealed that PARIS-overexpressing cells proliferated slower than control cells (Fig. 2c). Next, we determined whether cell death contributes to decreased differentiation of PARIS-overexpressing cells. Thus, control and PARIS-overexpressing C2C12 cells were subjected to flow cytometry for Annexin-V-fluorescein isothiocyanate (FITC) and propidium iodide (PI). As a control, C2C12 cells were treated with hydrogen peroxide and 71% cells were positive for Annexin-V, whereas control and PARIS-overexpressing C2C12 cells had 8.2% and 9.8%, respectively (Supplementary Fig. 2a). In addition, cleaved Caspase3 levels were not different between control and PARIS-overexpressing cells (Supplementary Fig. 2b). Thus, PARIS suppresses the proliferation of myoblasts without affecting cell death.

### PARIS triggers cellular senescence

As PARIS overexpression affected both myoblast proliferation and differentiation, we examined cellular senescence in PARIS-overexpressing cells. Approximately 12% of C2C12/PARIS cells were positive for SA-β-Gal staining with flat morphology, whereas few control cells were SA-β-Gal-positive (Fig. 2d, e), suggesting that PARIS triggers cellular senescence. To verify, PARIS expression was assessed in cellular senescence of mouse embryonic fibroblasts (MEFs). Freshly isolated MEFs were passaged every 2 days and examined the proliferation and senescent phenotypes. At passage 4 (P4), MEFs grew substantially slower and the proliferation was blunted at P7 (Supplementary Fig. 3a). MEFs at P7 showed senescence as indicated by the SA-β-Gal positivity when compared with unstained MEF at P2 (Supplementary Fig. 3b, c). In addition, the expression of senescence-associated secretory phenotype (SASP) genes (*IL6*, *Igfbp5*, *Igfbp7*, and *TNF-α*) and p21 was significantly increased in MEFs at P7 (Supplementary Fig. 3d). PARIS transcript levels increased gradually in MEFs with passaging (Supplementary Fig. 3e). The protein levels of PARIS and p21 were greatly elevated in MEFs at P5 and P6 (Supplementary Fig. 3f, g). MEFs at P7 were subjected to immunostaining for PARIS and a proliferation marker Ki67 (Supplementary Fig. 3h, i). The majority of cells were positive for either PARIS or Ki67 and only very low proportion of cells showed double positivity for them. To further examine whether PARIS upregulation is involved in cellular senescence triggered by DNA damage, MEFs were treated with doxorubicin (DOX) for 4 days to induce cellular senescence, as evident by SA-β-gal staining and the flat morphology (Supplementary Fig. 4a, b). The Dox treatment greatly elevated PARIS protein levels compared with the control treatment (Supplementary Fig. 4c). Similarly, the transcript levels of PARIS and p21 were significantly upregulated in DOX-treated cells, compared with the control (Supplementary Fig. 4d). Thus, the upregulation of PARIS occurs concomitantly with cellular senescence of MEFs.

Next, control or PARIS-transfected C2C12 cells were subjected to immunostaining for p-γH2AX, a marker for DNA damage, and GL13, a novel chemical analog is highly lipophilic and binds to lipofuscin^20^, associated with cellular senescence. PARIS-transfected C2C12 cells exhibited elevated p-γH2AX foci compared with control cells (Fig. 2f, g). Similar to the SA-β-Gal staining, PARIS-overexpressing cells showed GL13-positive staining, whereas PARIS-negative cells tend to be negative for GL13 (Supplementary Fig. 5). The expression of SASP genes, *TNF-α*, *interleukin-1α*, and *Igfbp5*, was elevated in PARIS-overexpressing myoblasts compared with control cells (Fig. 2h). In addition, the immunoblotting analysis revealed that PARIS-overexpressing cells had increased levels of cell cycle inhibitors, p21, p27, and p53, compared with pCMV-transfected cells (Fig. 2i, j). In addition, the immunostaining for p21 and PARIS indicated that about 23% of PARIS-positive cells were positive for p21 (Fig. 2k, l). Taken together, PARIS upregulation elicits cellular senescence in myoblasts and fibroblasts.

### PARIS elevates the expression of genes related to cytokine signaling and cell cycle arrest

**Fig. 3:**
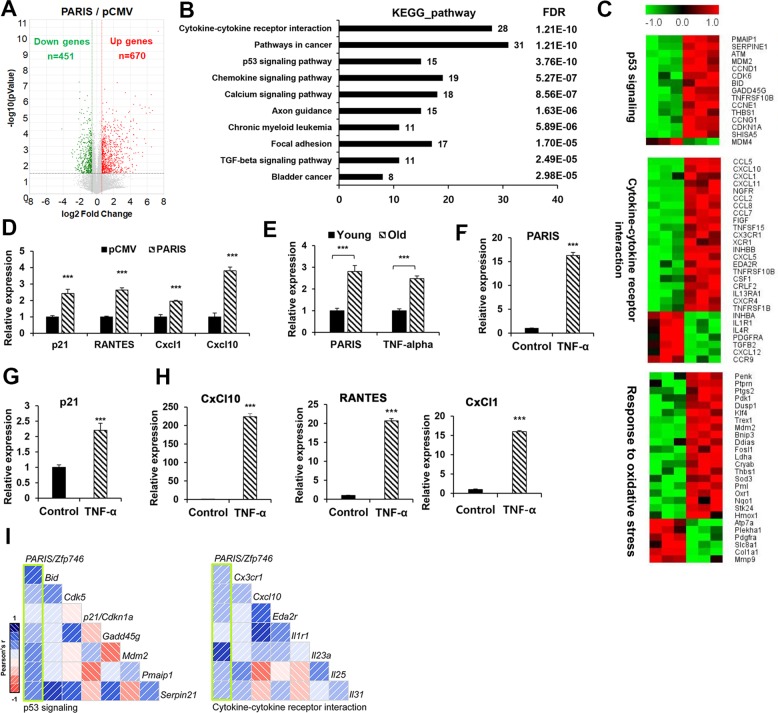
PARIS overexpression elevated genes related to cytokine signaling and cell cycle inhibition in myoblasts. **a** Volcano plot of gene expression presents the differential expression between pCMV- and pCMV-PARIS-overexpressing C2C12 cells (*n* = 3, each). Cut-off criteria is fold change 1.5 and *P* < 0.05. **b** KEGG pathway analysis for the RNA-sequencing data shown in **a**. Top ten ranks are presented. Cut-off criteria are *p* < 0.05 and FDR *q*-value < 0.05. **c** Heat map representing the relative mRNA expression levels in pCMV-PARIS-overexpressing C2C12 cells compared with the control cells. **d** qRT-PCR analysis for mRNA expression of *p21*, *RANTES*, *Cxcl1*, and *Cxcl10* in pCMV- and pCMV-PARIS-overexpressing C2C12 cells. The values were normalized to the level of an endogenous control *L32* (three sets per group). **e** qRT-PCR analysis for the expression of *PARIS* and *TNF-α* in EDL muscles from young (6 months) and old (26 months) mice (*n* = 3 per group). **f**–**h** qRT-PCR analysis for mRNA expression of the indicated genes in C2C12 cells with mock or TNF-α (20 ng/ml) treatment for 6 h (three sets per group). **i** Correlograms showing Pearson’s *r* between *PARIS* and genes of *p53* signaling and cytokine–cytokine receptor interaction in the skeletal muscle precursor cells of 2-, 12-, and 24-month-old mice (*n* = 14, NIH NCBI GEO number GSE50821), with the depth of the shading proportional to the magnitude of the correlation. The positive and negative correlations represented in blue and red, respectively. Significant difference was determined by Student *t*-test (***p < 0.001).

Next, we have examined the global gene expression in control or PARIS-overexpressing myoblasts. Genes (1121, 1.5-fold, *p* < 0.05) were significantly altered in PARIS-overexpressing cells (Fig. 3a). The Kyoto Encyclopedia of Genes and Genomes (KEGG) pathway and reactome analysis were performed and the top ten ranked signaling pathways with the cut-off criteria of *p*-value (<1.6e − 6) and false discovery rate (<0.05) are shown in Fig. 3b and Supplementary Fig. 6. The KEGG pathway and Gene Ontology analysis indicated that genes involved in KEGG_p53 signaling (15 genes), KEGG_cytokine-cytokine receptor interaction (28 genes), and GO_response to oxidative stress (26 genes) were significantly altered as depicted in a heat map (Fig. 3b, c). Among genes related to p53 signaling, p21 was increased about 1.6-fold. Furthermore, the reactome analysis revealed PARIS-dependent alteration in genes related to G1 damage response signaling (Supplementary Fig. 6), likely reflecting the cellular senescence. The quantitative reverse-transcriptase PCR (qRT-PCR) analysis confirmed that PARIS-overexpressing myoblasts expressed higher levels of p21 (~2.5-fold) and cytokines such as RANTES (Ccl5), Cxcl1, and Cxcl10 (~2.5–4-fold) (Fig. 3d). These data suggest that PARIS overexpression causes alteration in inflammatory responses. Pro-inflammatory cytokines such as tumor necrosis factor (TNF)-α have been implicated in muscle atrophy related to cancer cachexia or aging^21,22^. Consistently, TNF-α levels were significantly elevated in Extensor Digitorum Longus (EDL) muscles from 26-month-old (old) mice compared with 6-month-old (young) control muscles (Fig. 3e). PARIS expression was also greatly increased in old muscles, compared with the young muscles. This correlation made us to further examine the relationship between TNF-α and PARIS in myoblasts. C2C12 cells were treated with TNF-α (20 ng/ml) for 24 h and the expression of PARIS, p21, and other cytokines was assessed (Fig. 3f–h). TNF-α treatment greatly enhanced the level of PARIS about 16-fold and p21 about 2.3-fold. The expression of RANTES, Cxcl1, and Cxcl10 was greatly increased in TNF-α-treated cells compared with the control cells. The close examination of previously published transcriptomes of the skeletal muscle progenitor cells of 2-, 12-, and 24-month-old mice (*n* = 14, NIH NCBI GEO number GSE50821) revealed a positive correlation between PARIS, genes of p53 signaling, and cytokine–cytokine receptor interaction, similar to our current analysis in PARIS-overexpressing myoblasts (Fig. 3i). Taken together, PARIS overexpression causes alterations in inflammatory responses, response to oxidative stress, and p53 signaling related to cellular senescence.

### PARIS triggers oxidative stress response and FoxO1 accumulation in myoblasts

**Fig. 4:**
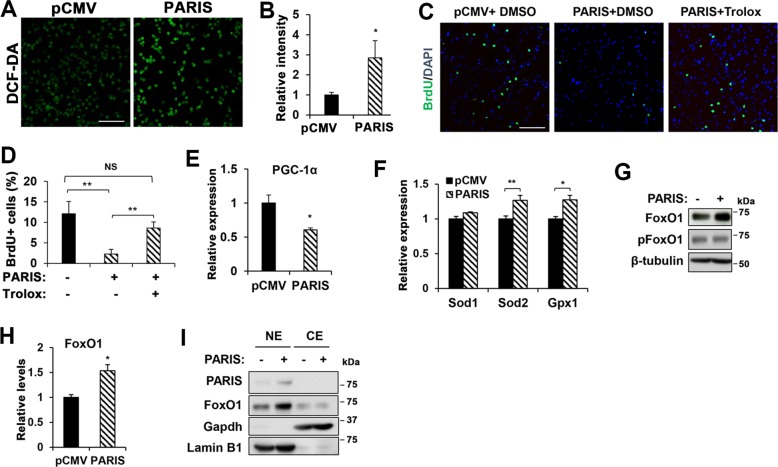
PARIS overexpression caused oxidative stress and enhanced FoxO1 level. **a** ROS generation was determined by treating 100 μM CM-H_2_DCFDA to pCMV- or pCMV-PARIS-overexpressing C2C12 cells. Scale bar = 100 μm. **b** Fluorescence intensities were quantified by ImageJ (four fields per group). **c** BrdU incorporation assay was performed with pCMV- or pCMV-PARIS-overexpressing C2C12 cells after treating mock (PBS) or 20 μM Trolox for 24 h. Scale bar = 100 μm. **d** The percentage of BrdU-positive cells (*n* = 4 per group, 276 ~ 301 (pCMV), 177 ~ 294 (PARIS), and 192 ~ 284 (PARIS + Trolox) cells per field were counted, respectively). **e** qRT-PCR analysis for mRNA expression of *PGC-1α* in pCMV- or pCMV-PARIS-overexpressing C2C12 cells (3 sets per group). (**f**) qRT-PCR analysis for mRNA expression of *Sod1*, *Sod2*, and *Gpx1* in pCMV- or pCMV-PARIS-overexpressing C2C12 cells (three sets per group). **g** Immunoblotting of FoxO1 and phospho-FoxO1 in pCMV- or pCMV-PARIS-overexpressing C2C12 cells. **h** Relative protein level of FoxO1 (three sets per group). **i** Nuclear extraction was performed with pCMV- or pCMV-PARIS-overexpressing C2C12 cells. Nuclear (NE) or cytosolic extracts (CE) were subjected to immunoblotting for FoxO1 and PARIS. Gapdh and Lamin B1 were selected as a cytosolic and nuclear marker, respectively. Significant difference was determined by Student *t*-test (**b**, **e**, **f**, **h**) and ANOVA (**d**) (*p < 0.05, **p < 0.01).

As oxidative stress is one of the major trigger for cellular senescence, we next examined the effect of PARIS overexpression on oxidative stress in myoblasts. Control and PARIS-overexpressing C2C12 cells were subjected to staining with 2’,7’-dichlorofluorescin diacetate (DCF-DA) to detect the cellular reactive oxygen species (ROS). Unlike the control cells, PARIS-overexpressing cells showed a robust staining for DCF-DA with a threefold increase (Fig. 4a, b). To examine the role of oxidative stress in PARIS-triggered cell cycle arrest, control and PARIS-overexpressing myoblasts were treated with an antioxidant 6-hydroxy-2,5,7,8-tetramethylchroman-2-carboxylic acid (Trolox, 20 μM) for 24 h followed by BrdU incorporation for 10 min and immunostaining. As previously shown, vehicle dimethyl sulfoxide-treated PARIS-overexpressing myoblasts exhibited greatly reduced BrdU incorporation, whereas Trolox treatment significantly restored the proliferation of PARIS-overexpressing cells almost to the level of control cells (Fig. 4c, d). Thus, oxidative stress might be the cause for the impaired proliferation of PARIS-overexpressing myoblasts. Previous studies have shown that PARIS acts as a repressor for PGC-1α, which can be induced by oxidative stress and regulates the induction of antioxidant response genes^17,23^. It is conceivable that PARIS-mediated PGC-1α repression might reduce the expression of antioxidant genes, such as Superoxide dismutase 1 (*SOD1*), *SOD2*, and Glutathione peroxidase (*GPX*)^23–26^, contributing to cellular senescence. However, the qRT-PCR analysis revealed that *PGC-1α* expression was significantly reduced in PARIS-overexpressing myoblasts, whereas the expression of *SOD1*, *SOD2*, and *GPX1* was mildly increased compared with the control levels in response to oxidative stress (Fig. 4e, f). Thus, it is concluded that the suppression of these antioxidant genes is not the major target of PARIS.

FoxO transcription factors are activated by multiple stresses including oxidative stress and the dysregulation of FoxOs has been implicated in cellular senescence through induction of p21 and p27^15,27^. Considering the fact that PARIS and FoxOs can modulate gene expression through IRE, we have examined the involvement of FoxOs in PARIS-induced cellular senescence. PARIS-overexpressing cells expressed higher levels of FoxO1 proteins, relative to control cells while the phosphorylated form of FoxO1 was unchanged (Fig. 4g, h), suggesting that the growth factor signaling-mediating FoxO1 inhibition occurs normally in PARIS-overexpressing cells. The phosphorylation of FoxO1 induces its cytoplasmic localization thereby inhibiting its transcriptional activity^12,13^. Consistently, the level of cytoplasmic FoxO1 was unchanged in PARIS-overexpressing myoblasts, whereas nuclear FoxO1 levels were elevated (Fig. 4i). As PARIS overexpression did not enhance *FoxO1* mRNA levels in myoblasts (Supplementary Fig. 7a), it is concluded that PARIS overexpression elevates FoxO1 protein stability rather than the expression of FoxO1. Taken together, PARIS overexpression causes excessive oxidative stress leading to FoxO1 protein accumulation.

### Depletion of FoxO1 attenuates PARIS-induced senescence in myoblasts

**Fig. 5:**
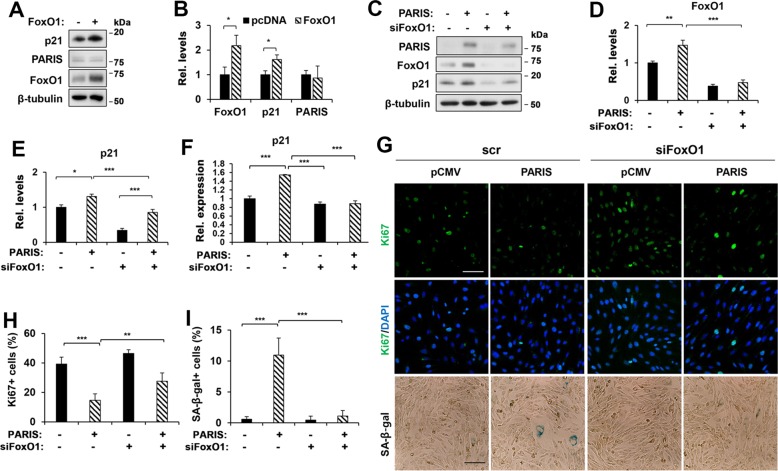
Cellular senescence caused by PARIS was prevented by FoxO1 depletion in myoblasts. **a** Immunoblot analysis for p21, PARIS, and FoxO1 in pcDNA- or pcDNA-FoxO1-overexpressing C2C12 cells. **b** The relative protein level of FoxO1, p21 and PARIS (three sets per group). **c** Immunoblotting for p21, PARIS, and FoxO1 in C2C12 cells, which were co-transfected with Scrambled (Scr) or FoxO1 siRNA (siFoxO1) and with pCMV or pCMV-PARIS plasmid. **d**, **e** Relative protein levels of FoxO1 and p21 (three sets per group). **f** qRT-PCR analysis for p21 in C2C12 cells, which were co-transfected with Scrambled or siFoxO1 and with pCMV or pCMV-PARIS plasmid (three sets per group). **g** Immunostaining of Ki67 (green) in C2C12 cells, which were transfected with the indicated plasmid and/or siRNA. The transfected C2C12 cells were also subjected to SA-β-gal staining. Scale bar = 100 μm (upper), 50 μm (bottom). **h** Quantification of Ki67-positive cells (*n* = 5 per group, 76 ~ 128 (pCMV + Scr), 61 ~ 130 (PARIS + Scr), 62 ~ 76 (pCMV + siFoxO1), and 51 ~97 (PARIS + siFoxO1) cells per field were counted, respectively). **i** Quantification of SA-β-gal-positive cells (*n* = 4 per group, 90 ~ 119 (pCMV + Scr), 51 ~ 72 (PARIS + Scr), 67 ~ 76 (pCMV + siFoxO1), and 68 ~ 91 (PARIS + Scr) cells per field were counted, respectively). Significant difference were determined by Student’s *t*-test (**b**) and ANOVA (**d**, **e**, **f**, **h**, **i**) (*p < 0.05, **p < 0.01, ***p < 0.001).

Next, the effect of FoxO1 overexpression on p21 expression was examined in C2C12 myoblasts. In consistent with the previous report^15,27,28^, FoxO1 overexpression upregulated p21 levels in C2C12 myoblasts, without elevating PARIS levels (Fig. 5a, b). Perturbation in autophagy is also implicated in cellular stress leading to cell death or senescence^5,29–31^. Thus, we have examined the autophagy response in control and PARIS-overexpressing cells. Control and PARIS-overexpressing cells were cultured in normal growth medium or 0.1% fetal bovine serum (FBS) containing medium for 24 h and assessed autophagy by the level of lipidated LC3 (LC3-II) and p62. PARIS overexpression did not alter greatly autophagy (Supplementary Fig. 7b). In addition, we have examined the expression of autophagy-related genes, *Atrogin1*, *LC3*, *Beclin*, *Atg5*, *Ulk1*, and *Bnip3* in cells with either PARIS overexpression or depletion (Supplementary Fig. 7c, d). The expression of autophagy-related genes did not alter significantly in relation with different PARIS levels. These data suggest that FoxO1 activation in response to PARIS does not alter autophagy. It can be further hypothesized that PARIS regulates p21 levels through FoxO1 leading to myoblast senescence. To test this hypothesis, control or PARIS-overexpressing C2C12 cells were transfected with scrambled or FoxO1 siRNAs. Three different siFoxO1 were tested and siFoxO1-2 was used in further study due to efficient knockdown (Supplementary Fig. 8). FoxO1 depletion reduced the expression of p21 compared with the control cells (Fig. 5c–f). In consistent with the previous data, PARIS overexpression led to upregulation of FoxO1 and p21 protein and FoxO1 depletion in PARIS-overexpressing cells reduced both p21 mRNA and protein levels. Unlike the complete abrogation of p21 mRNA induction, p21 protein levels were less efficiently suppressed by FoxO1 depletion suggesting for a posttranslational mechanism to modulate p21 proteins in PARIS-overexpressing myoblasts. The effect of FoxO1 depletion on proliferation of PARIS-overexpressing cells was further examined by immunostaining for Ki67 and SA-β-Gal staining (Fig. 5g–i). The decrease in Ki67-positive cell proportion in PARIS-overexpressing cells was partially but significantly attenuated by FoxO1 depletion. In contrast, the increase in SA-β-Gal-positive cell proportion was significantly decreased by FoxO1 depletion. Taken together, PARIS overexpression augments excessive oxidative stress and FoxO1 activity, contributing to cellular senescence in myoblasts.

### PARIS enhances the recruitment of FoxO1 to the *p21* promoter region

**Fig. 6:**
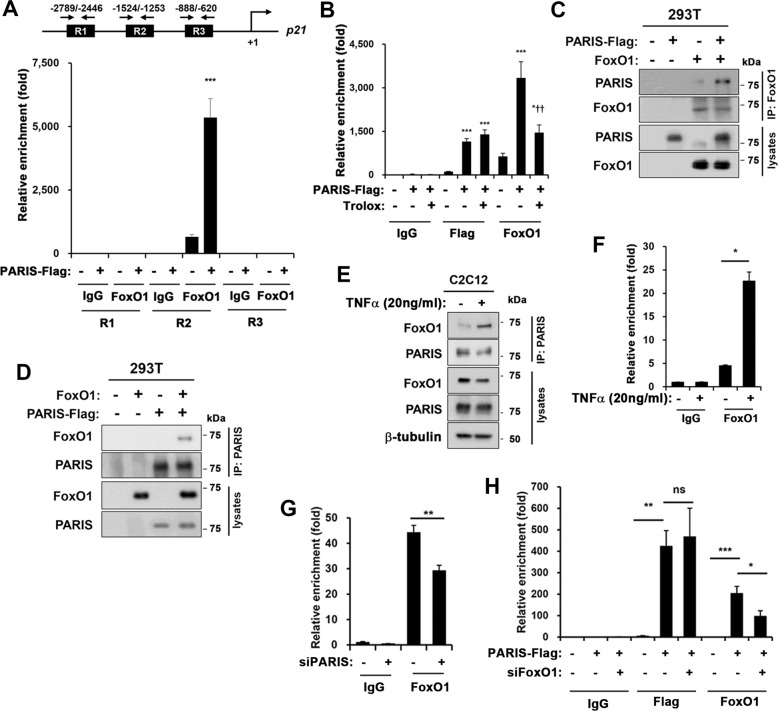
PARIS enhanced FoxO1 binding capacity of the *p21* promoter region. **a** Putative FoxO1 or PARIS binding sites in the *p21* (Cdkn1a) promoter. These proximal (-888/-620; R3), middle (-1524/-1253; R2), and distal (-2789/-2446; R1) regions contain IRE. Control or PARIS-Flag-overexpressing C2C12 cells were subjected to Chip assay with anti-FoxO1 antibody using three primer sets for R1-R3 regions in the *p21* promoter (three sets per group). **b** Control or PARIS-overexpressing C2C12 cells were treated with 20 μM of Trolox for 24 h in growth condition and subjected to Chip assay with anti-FoxO1 and Flag antibodies (three sets per group). **c**, **d** Immunoprecipitation assays in 293T cells expressing PARIS-Flag and FoxO1 with FoxO1 or PARIS antibodies. **e** Co-immunoprecipitation of FoxO1 with anti-PARIS antibody in C2C12 cells treated with TNF-α (20 ng/ml) for 24 h. **f** C2C12 cells were treated with 20 ng/ml TNF-α for 6 h and subjected to ChIP assay with anti-FoxO1 or IgG antibodies. Relative enrichment was measured by a ratio of control and PARIS-Flag-overexpressing cells in relation to IgG control (three sets per group). **g** Control or siPARIS-expressing C2C12 cells were subjected to ChIP assay with anti-FoxO1 or IgG antibodies (three sets per group). **h** PARIS-Flag and/or siFoxO1-expressing C2C12 cells were subjected to ChIP assay with anti-FoxO1 and Flag antibodies. Relative enrichment was measured by a ratio of each values in relation to IgG control (three sets per group). Statistical values were determined by ANOVA (*n* = 3, *p < 0.05, *** *p* < 0.001, ^††^*p* < 0.01).

As PARIS overexpression resulted in nuclear FoxO1 accumulation, we have assessed the recruitment of FoxO1 and PARIS in three IRE-containing regions (-888/-620; -1524/-1253; -2789/-2446) of the upstream regulatory region in the *p21* promoter (Fig. 6a). Interestingly, the chromatin immunoprecipitation (ChIP) assay revealed the specific recruitment of FoxO1 to the region -1524/-1253 and the PARIS overexpression elicited a robust enrichment of FoxO1 to this region. In consistent with the rescuing effects of Trolox on cell proliferation shown in Fig. 4c, the FoxO1 enrichment to this promoter region was blunted by Trolox treatment (Fig. 6b). Unfortunately, we were unable to immunoprecipitate with the commercially available PARIS antibodies tested in this study. Thus we have used the anti-Flag antibody to precipitate the overexpressed Flag-tagged PARIS on chromatin (Fig. 6b). The ChIP experiment with anti-Flag antibody showed a specific enrichment of PARIS in the same region of the *p21* promoter like FoxO1, suggesting that PARIS might be co-recruited with FoxO1. Unlike FoxO1 recruitment to the *p21* promoter, PARIS recruitment was not affected by the Trolox treatment. These data suggest that the FoxO1 recruitment to the p21 regulatory region is triggered by oxidative stress. We then have examined the potential physical interaction between PARIS and FoxO1 in 293T cells (Fig. 6c). PARIS and FoxO1 were reciprocally co-immunoprecipitated when overexpressed. Based on the effect of TNF-α on PARIS and p21 upregulation, C2C12 cells were treated with TNF-α and analyzed for the interaction between PARIS and FoxO1 (Fig. 6d). The treatment of TNF-α elevated the interaction between FoxO1 and PARIS, likely contributing to p21 upregulation (Fig. 6e, f). To assess the effect of PARIS depletion on FoxO1 recruitment to *p21* promoter region, control or siPARIS-expressing C2C12 cells were subjected to ChIP assay. FoxO1 recruitment was significantly reduced by PARIS depletion (Fig. 6g). Next the effect of FoxO1 depletion on PARIS recruitment to *p21* promoter was examined. The depletion of FoxO1 did not affect the enrichment of PARIS to *p21* promoter, whereas the PARIS-mediated FoxO1 recruitment was significantly reduced by FoxO1 depletion (Fig. 6h). Taken together, these data suggest that PARIS and FoxO1 are co-recruited to *p21* promoter in response to oxidative stress.

In addition to PARIS/FoxO1/p21 axis, the global gene expression profile suggested the potential involvement of p53 signaling in PARIS-mediated p21 expression. As overexpression in myoblasts elevated p53 proteins (Fig. 2j), there was no change in p53 mRNA levels observed from the RNA-sequencing data (Fig. 4a). PARIS overexpression appears to regulate p53 protein accumulation, likely related to oxidative stress observed in PARIS-overexpressing myoblasts. Thus, we examined the effect of p53 depletion on p21 expression in response to PARIS overexpression. C2C12 cells were transfected with scrambled or three different p53 siRNAs (Supplementary Fig. 9a). Among the three different sip53, sip53#3 was used in further study. The p21 expression was significantly decreased in both control and PARIS-overexpressing myoblasts (Supplementary Fig. 9b, c). In conclusion, these data suggest that PARIS induces p21 expression through p53 and FoxO1 leading to impaired cell proliferation and cellular senescence.

## Discussion

Oxidative stress in muscle stem cells has been linked with decreased regenerative capacity accompanied by cell death or senescence^5,32^. In this study, we demonstrate that PARIS overexpression induced cellular senescence with elevated DNA damage accompanied by impaired proliferation and differentiation of myoblasts. Interestingly, PARIS overexpression elevated ROS levels and Trolox treatment restored proliferation of PARIS-overexpressing cells, suggesting that PARIS overexpression perturbs the control mechanism of redox homeostasis. Previously PARIS has been associated with Parkinson’s diseases^17^. PARIS is targeted by Parkin to be degraded and in Parkinson’s disease the level of PARIS increases leading to repression of PGC-1α and mitochondrial dysfunction^17,33^. PGC-1α levels are critical for the expression of antioxidant genes, such as *SOD1*, *SOD2*, and *GPx1*^26^. PGC-1α-deficient fibroblasts and mice are more sensitive to oxidative stress^34^. PARIS-overexpressing myoblasts exhibited decreased PGC-1α transcript however the levels of *SOD1*, *SOD2*, and *GPx1* genes were rather slightly increased. Thus, it can be concluded that the induction of antioxidant gene expression by PGC-1α might not be the major defect in PARIS-overexpressing cells. However, we cannot entirely rule out the partial contribution of PGC-1α in PARIS-elicited cellular senescence.

FoxOs have been implicated in the control of diverse cellular processes, including autophagy, stem cell quiescence, and senescence^10,12^. FoxO1 activates the expression of cell cycle inhibitors, p21 or p27, through interaction to FoxO-response element in the promoter region of these genes^15,27,35^. PARIS overexpression upregulated the levels of p21 and p27 proteins likely contributing to cell cycle inhibition and senescent phenotype of myoblasts. FoxO1 and p53 hyperactivation seems to be linked with p21 induction and cellular senescence of PARIS-overexpressing myoblasts. PARIS-overexpressing cells expressed higher levels of FoxO1 and p53 proteins without upregulated transcription of these genes. Similar to PARIS overexpression, FoxO1 overexpression increased p21 levels. However, PARIS levels were unchanged by FoxO1 overexpression, suggesting that FoxO1 might be acting downstream of PARIS. In consistent with this assumption, FoxO1 depletion in PARIS-overexpressing cells reduced p21 levels and restored myoblast proliferation. In addition, FoxO1 depletion in PARIS-overexpressing cells reduced DNA damage response and senescent phenotypes. Thus, these data suggest that the increased PARIS levels might perturb the redox homeostasis through yet to be identified mechanism and this will elevate FoxO1 and p53 levels contributing to cell cycle inhibition and senescence.

PARIS seems to regulate FoxO1 protein stability or nuclear localization. However, the exact mechanism is currently unclear. As the level of phosphorylated FoxO1 did not change in PARIS-overexpressing cells, it appears that PARIS does not interfere with AKT-mediated FoxO1 phosphorylation leading to cytosolic localization and degradation of FoxO1^12,28^. One possible mechanism might be through a physical interaction and co-recruitment of PARIS and FoxO1 to the *p21* promoter region between -1253 and -1524. Furthermore, PARIS overexpression enhanced the recruitment of FoxO1 to this region, which was attenuated by the Trolox treatment. Unlike FoxO1, the Trolox treatment did not affect the level of PARIS recruited to the *p21* promoter region. Thus, it can be concluded that FoxO1 recruitment to the *p21* promoter region is dependent on cellular oxidative stress. Recently, FoxO1 has been shown to induce cellular senescence in ovarian cancer cells by the co-recruitment with progesterone receptor to the *p21* promoter region^36^. Similarly, FoxO1 appears to be co-recruited with PARIS in the *p21* promoter region containing IRE. Due to the technical difficulty, our data are based on the PARIS overexpression study. Thus, we need to verify the recruitment of endogenous PARIS under oxidative stress condition, which might provide more accurate physiological role of PARIS in cell senescence in response to oxidative stress.

Although the endogenous signaling controlling PARIS expression is currently unclear, PARIS levels were greatly elevated along with TNF-α in aged muscles, relative to young muscles. Interestingly, inflammatory cytokines, such as TNF-α evoke generation of ROS and elevated inflammatory signals have been closely linked with muscle aging and decreased regenerative capacity^21,22^. Thus, it is well likely that PARIS is involved in oxidative stress related to muscle aging through corroborating with TNF-α. In support of this hypothesis, the expression of PARIS and p21 was augmented by TNF-α treatment in myoblasts. Further studies will be required to elucidate the role of PARIS in TNF-α mediated inflammatory responses and muscle stem cell dysfunction. Our current study demonstrates a suppressive role of PARIS in muscle regeneration through perturbation of redox homeostasis. Similar to the strategy proposed to treat Parkinson’s diseases, PARIS upregulation needs to be prevented to maintain muscle regenerative capacity. Consistent with this notion, Parkin overexpression has been shown to prevent muscle atrophy related to muscle aging^37^. Considering the fact that Parkin can suppress PARIS, the effects observed by Parkin overexpression could be at least partly contributed by PARIS reduction. Thus, PARIS might represent an attractive target to prevent aging-related diseases, such as Parkinson’s diseases as well as sarcopenia.

## Materials and methods

### Cell culture, counting, and transfection

C2C12 myoblasts, 293T cells, and MEF cultures were carried out as previously described^38,39^. C2C12 cells were cultured in Dulbecco modified Eagle medium (DMEM) containing 15% FBS, and 1× Penicillin–Streptomycin. To induce myogenic differentiation, cells were cultured in DMEM containing 2% horse serum at near confluence. MEFs were isolated from E13.5 embryos of C57BL/6 mice and cultured in DMEM containing 10% FBS and 1× Penicillin–Streptomycin. 10T1/2 cells were cultured in DMEM containing 10% FBS and 1× Penicillin–Streptomycin. For passaging MEFs, ~3 × 10^5^ cells were plated and counted 2 days later, to determine the growth rate. The numbers of viable cells, which were excluded from staining with Trypan blue were counted by using hemocytometer. For transfection, pCMV, pCMV-Flag-PARIS, pcDNA3, pcDNA3-FoxO1, or siRNAs were transfected to the corresponding cells with Lipofectamin 2000 (Invitrogen), according to manufacturer’s instructions. siRNA sequences are listed in Supplementary Table 1. Recombinant human TNF-α and Trolox were purchased from Abcam and Sigma-Aldrich, respectively.

### Immunostaining, immunoblotting, and immunoprecipitation

Immunocytochemisty were performed as previously described^39,40^. The fixed cells were incubated with primary antibodies including anti-PARIS (Millipore, MABN476), anti-PARIS (Abcam, ab130867), anti-MHC (DSHB, MF20), anti-Ki67 (Abcam, ab15580), anti-p21 (Santa Cruz, sc-471), anti-phospho-γH2AX (Ser139) (Cell Signaling, 9718), anti-Biotin (Jakson, 115-065-146), and anti-Flag (Antibody Frontier, LF-PA20169) antibody. Then, incubation with Alexa Fluor^®^ 488 goat anti-mouse antibody or Alexa Fluor^®^ 555 goat anti-rabbit antibody (Invitrogen) was performed for 1 h at room temperature. Images were captured by Zeiss LSM-510 Meta confocal microscope and Nikon ECLIPSE TE-2000U microscope. NIS-Elements F software (Nikon) and ImageJ software were employed to quantify for images. To evaluate ROS production in cells, cells were treated with 100 μM CM-H_2_DCFDA (Invitrogen) for 30 min. Fluorescence images in live cells were captured using Zeiss LSM-510 Meta confocal microscope and quantified by ImageJ.

Immunoblot analysis were performed as described previously^40^. Cell lysates were prepared by RIPA buffer (Invitrogen) and separated by SDS-polyacrylamide gel electrophoresis (PAGE). The blots were incubated with primary antibodies including anti-PARIS (Millipore, MABN476), anti-Myogenin (DSHB, F5D), anti-MHC (DSHB, MF20), anti-Caspase3 (Santa Cruz, sc-7148), anti-p21 (Santa Cruz, sc-471), anti-p27 (Abcam, ab7961), anti-p53 (Santa Cruz, sc-6243), anti-FoxO1 (Cell Signaling, 2880S), anti-phospho-FoxO1 (Ser256) (Cell Signaling, 9461S), anti-GAPDH (Antibody Frontier, LF-PA0018), anti-Lamin B1 (Abcam, ab16048), anti-PGC-1α (Calbiochem, ST1202), TnT (Abcam, ab10214), anti-MyoD (Santa Cruz, sc-760), anti-LC3 (Sigma-Aldrich, L8918), anti-p62 (Santa Cruz, sc-48402), and anti-β-tubulin antibody (Sigma-Aldrich, T5293). Then, samples were incubated with appropriate secondary antibodies conjugated with horseradish peroxidase and signals were detected by ECL (West Save).

Immunoprecipitation assays were carried out as previously described^41^. Cells for co-immunoprecipitation were lysed by extraction buffer containing 10 mM Tris-HCl (pH 7.4), 150 mM NaCl, 1 mM EDTA, 1% Triton X-100 and complete protease inhibitor cocktail (Roche). Then, samples were incubated with magnetic beads (Invitrogen) and anti-FoxO1 antibody at 4^o^C overnight. Samples were boiled and separated by SDS-PAGE. Subsequently, dissociated proteins were detection by anti-PARIS antibody.

### Quantitative RT-PCR

Total RNA from cells were isolated with Easy Blue (Intron), according to manufacturer’s instructions. After that, cDNA was synthesized using PrimeScript^TM^ cDNA synthesis kit (TaKaRa) according to the manufacturer’s instructions. qRT-PCR were performed by real-time PCR system with SYBR Premix ExTag^TM^ according to the manufacturer’s instructions (TaKaRa). Primer sequences are listed in Supplementary Table 2.

### Library preparation and RNA sequencing

For RNA sequencing, transfected confluent C2C12 cells were incubated in the medium containing 0.1% FBS for 24 h. Transcriptional profiles were analyzed by e-Biogen corporation. Libraries were prepared from total RNA using the SMARTer Stranded RNA-Seq Kit (Clontech Laboratories). The isolation of mRNA was performed using the Poly(A) RNA Selection Kit (LEXOGEN). The isolated mRNAs were used for the cDNA synthesis and shearing, following manufacture’s instruction. Indexing was performed using the Illumina indexes 1–12. The enrichment step was carried out using of PCR. Subsequently, libraries were checked using the Agilent 2100 bioanalyzer (DNA High Sensitivity Kit) to evaluate the mean fragment size. Quantification was performed using the library quantification kit using a StepOne RealTime PCR System (Life Technologies). High-throughput sequencing was performed as paired-end 100 sequencing using HiSeq 2500 (Illumina). For data analysis, mRNA-Seq reads were mapped using TopHat software tool in order to obtain the alignment file. Differentially expressed genes were determined based on counts from unique and multiple alignments using coverage in Bedtools. The RT (Read Count) data were processed based on Quantile normalization method using EdgeR within R (R development Core Team, 2016) using Bioconductor. The alignment files also were used for assembling transcripts, estimating their abundances and detecting differential expression of genes or isoforms using cufflinks. And we used the FPKM (fragments per kilobase of exon per million fragments) as the method of determining the expression level of the gene regions.

Transcriptomes of the skeletal muscle precursor cells of 2-, 12-, and 24-month-old mice were obtained from the publicly available data of the NIH NCBI Gene Expression Omnibus (*n* = 14, NIH NCBI GEO number GSE50821). Correlogram was generated using RStudio (R Consortium Inc, Boston, MA) as described in the previous study^42^.

### BrdU incorporation, cell apoptosis, and cellular senescence assay

For BrdU incorporation, 10 μM BrdU (Sigma-Aldrich, B5002) was treated to the cells in the dark for 10~15 min. The treated cells were fixed and permeabilized with 2 N HCl at 37 degrees for 30 min. After neutralizing with 0.1 M borate buffer (pH 8.5) for 12 min, cells were incubated with anti-BrdU antibody (Chemicon, MAB3222) at 4 °C during overnight. Then, cells were incubated with Alexa Fluor^®^ 488 goat anti-mouse antibody at room temperature for 1 h. Images were captured by Zeiss LSM-510 Meta confocal microscope and Nikon ECLIPSE TE-2000U microscope. NIS-Elements F software (Nikon) and ImageJ software were employed to quantify for images.

Apoptosis analysis was performed by staining with Annexin-V-FITC dye (Bio bud), according to manufacturer’s protocols. Dissociated C2C12 cells were incubated with Annexin-V-FITC dye for 15 min in cold PBS and then these cells were resuspended in fluorescence-activated cell sorting (FACS) buffer. Detection of Annexin-V-FITC (518 nm) and PI (620 nm) was performed by FACS (FACS Canto2), along with H_2_O_2_-treated positive and not stained negative controls.

To assess cellular senescence, SA-β-Gal assay was carried out by using SA-β-Gal assay kit (Cell signaling), according to manufacturer’s protocols. Briefly, fixed cells were stained with the provided pH 5.9 ~ 6.1 mixture containing β-gal overnight. GL13 (Sentragor) staining were performed by manufacturer’s protocols. Images of SA-β-Gal and GL13-positive cells were captured by Nikon ECLIPSE TE-2000U microscope.

### Cytoplasmic/nuclear fraction

Cells were incubated with cytoplasm extracts buffer containing 10 mM HEPES (pH 7.9), 0.08% Triton X-100, 50 mM NaCl, 0.5 M Sucrose, 0.1 mM EDTA, and proteinase inhibitor cocktail (Roche) on ice for 5 min and after centrifugation (2000 r.p.m. for 15 min), the supernatant was taken for cytoplasmic fraction. Following washing the pellet with Wash buffer (10 mM HEPES (pH 7.9), 10 mM KCl, 0.1 mM EDTA and proteinase inhibitor cocktail) twice, the pellet was lysed with Nuclear extract buffer containing 10 mM HEPES (pH 7.9), 500 mM NaCl, 0.1 mM EDTA, 0.1% NP40, and proteinase inhibitor cocktail on ice for 30 min and the supernatant was taken for nuclear fraction.

### Chromatin immunoprecipitation assay

ChIP analysis was carried out as previously described. Antibodies used in this study are as following; rabbit IgG (Millipore, 12-370), FoxO1 (Cell signaling, 2880 S) and Flag (Sigma-Aldrich, F3040). The primers used to amplify the *p21* promoter regions designated as R1 (-620/-888), R2 (-1253/-1524), and R3 (-2446/-2789) are listed in Supplementary Table 2.

### Statistical analysis

Statistical differences between two or multiple groups were analyzed by paired or unpaired two-tailed Student’s *t*-test or one-way analysis of variance test. All results are representative of three or more independent experimental sets. Data are expressed as means ± SD or ±SEM, as indicated in the figure legends. Differences were considered statistically significant as *p < 0.05, **p < 0.01, and ***p < 0.001.

## Supplementary information

Supplementary Figure 1

Supplementary Figure 2

Supplementary Figure 3

Supplementary Figure 4

Supplementary Figure 5

Supplementary Figure 6

Supplementary Figure 7

Supplementary Figure 8

Supplementary Figure 9

Table

Related Manuscript File

## Data Availability

All relevant data are included in the main article and Supplementary Figures. Additional information that support the findings of this study are available upon request from the corresponding author.
