# Supplementary figures and images for: ZNF746/PARIS overexpression induces cellular senescence through FoxO1/p21 axis activation in myoblasts
Source: Cell Death Dis. 2020 May 12;11(5):359. doi: 10.1038/s41419-020-2552-7 (PMC7217926; doi:10.1038/s41419-020-2552-7)

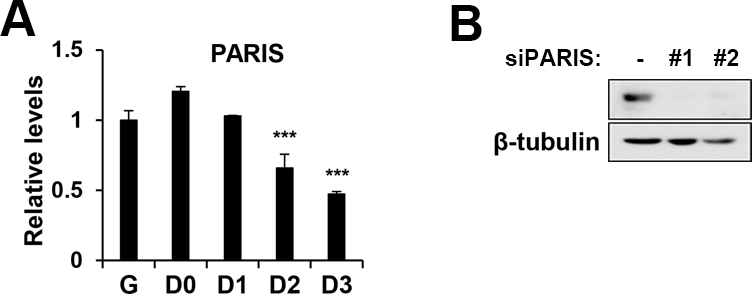

Supplement: Supplementary file 1 — Supplementary Figure 1 [file 41419_2020_2552_MOESM1_ESM.tif]

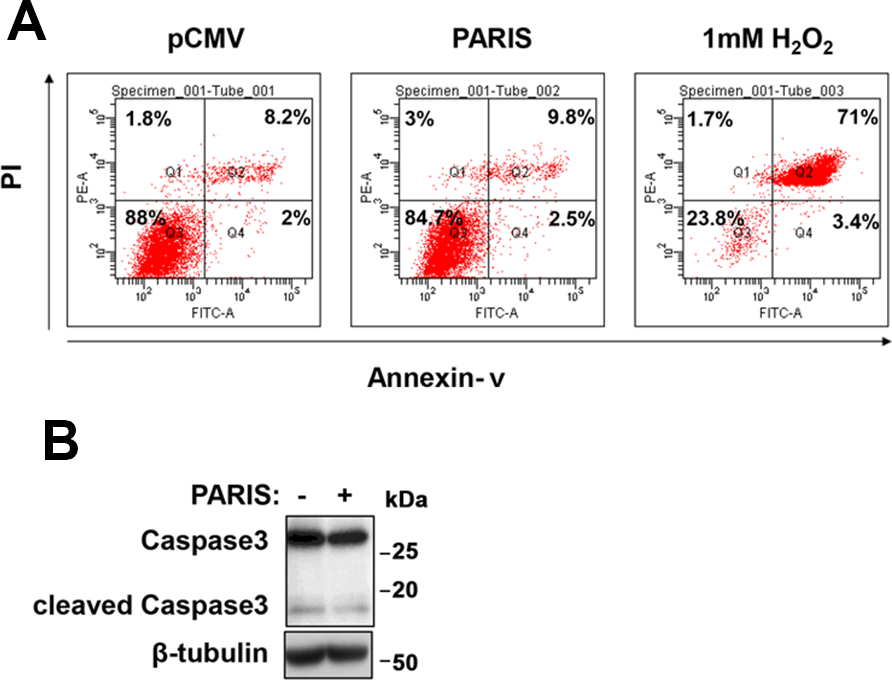

Supplement: Supplementary file 2 — Supplementary Figure 2 [file 41419_2020_2552_MOESM2_ESM.tif]

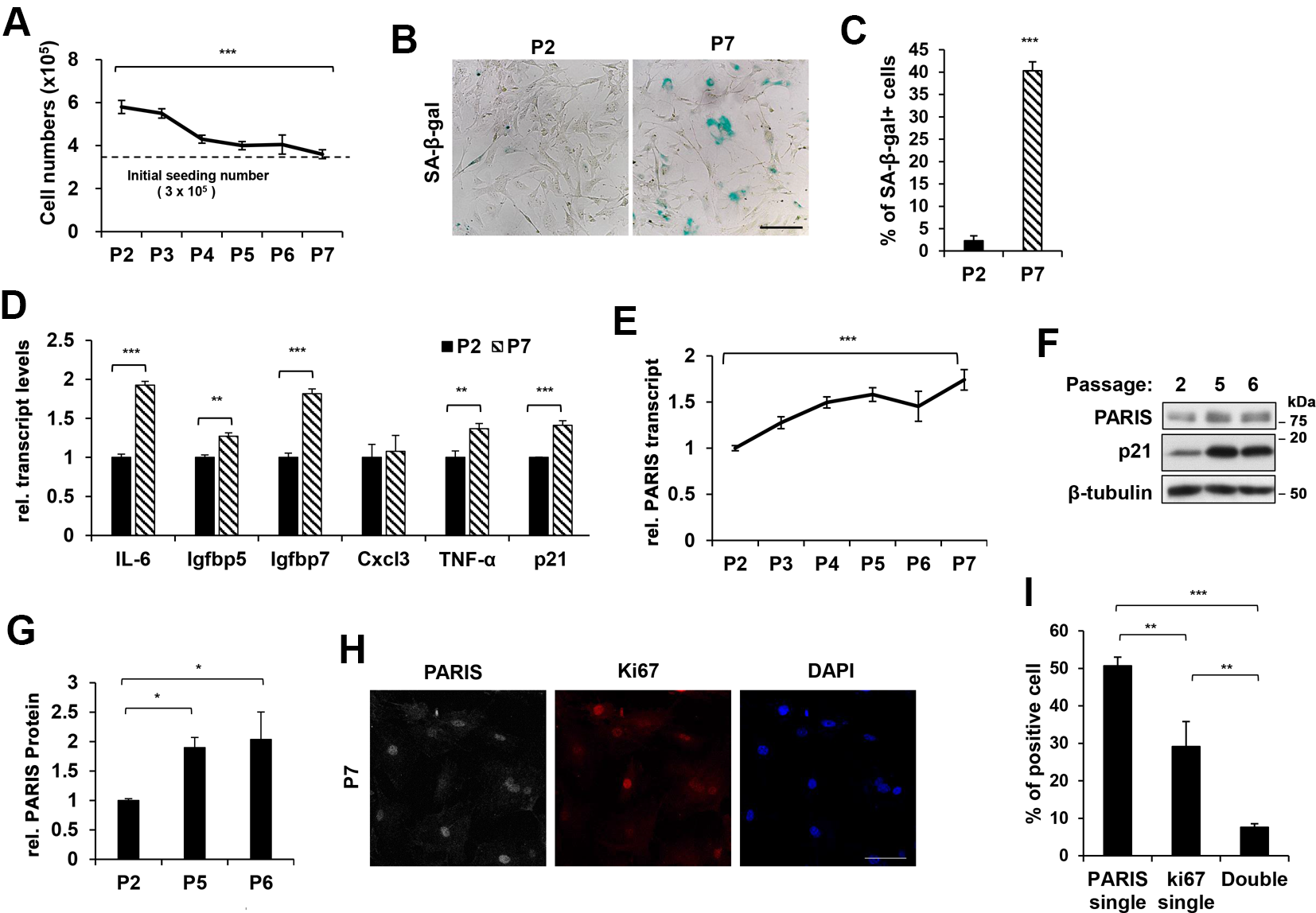

Supplement: Supplementary file 3 — Supplementary Figure 3 [file 41419_2020_2552_MOESM3_ESM.tif]

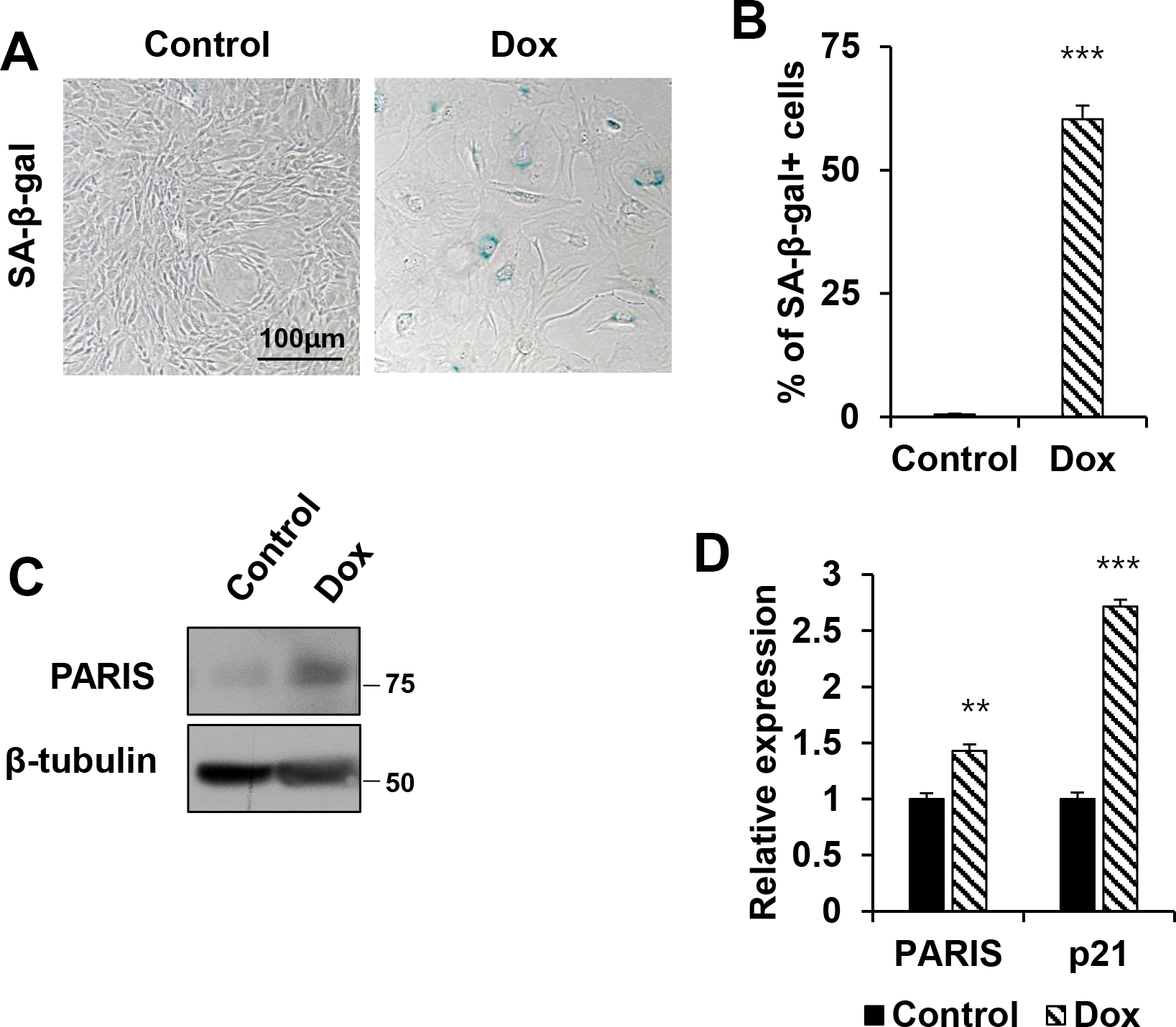

Supplement: Supplementary file 4 — Supplementary Figure 4 [file 41419_2020_2552_MOESM4_ESM.tif]

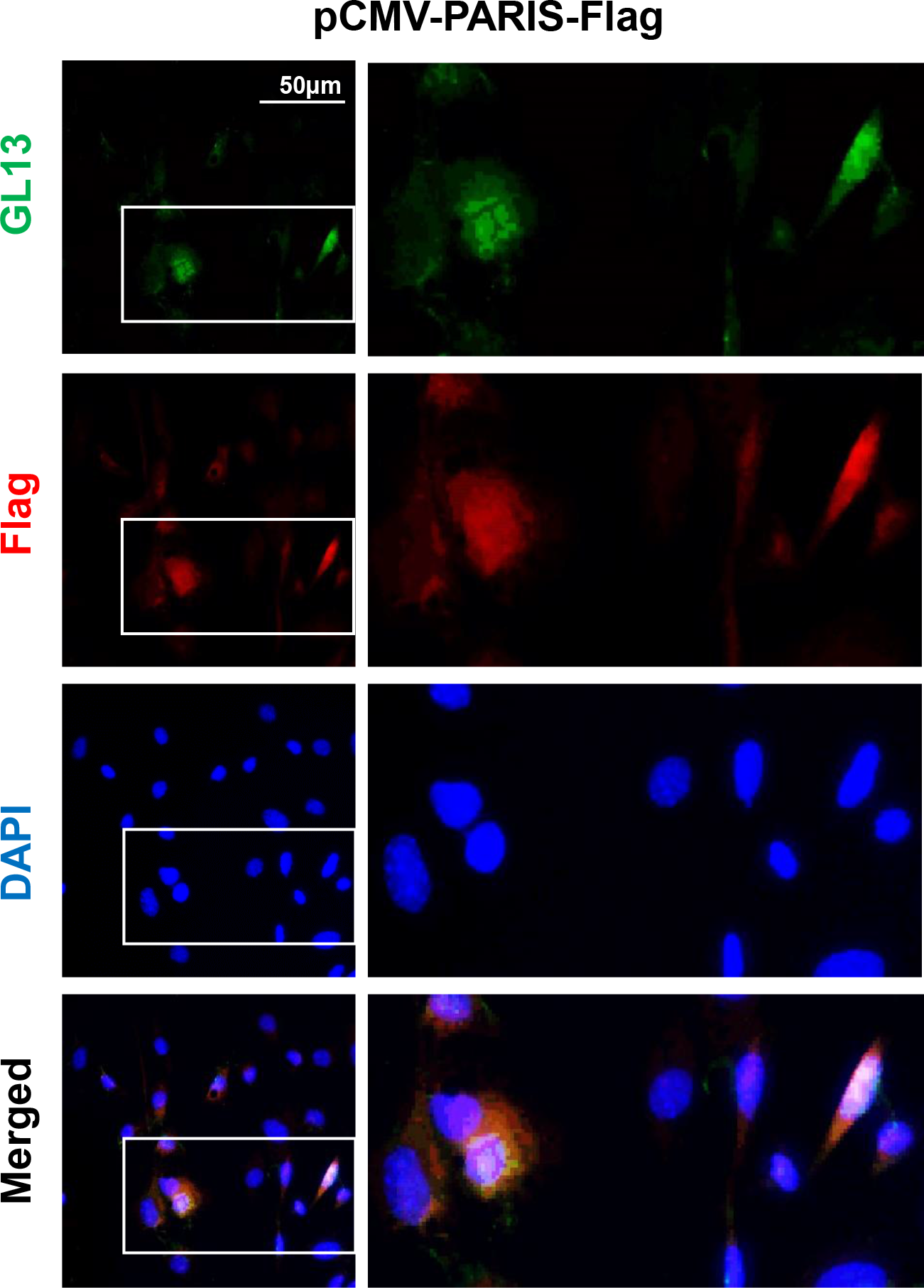

Supplement: Supplementary file 5 — Supplementary Figure 5 [file 41419_2020_2552_MOESM5_ESM.tif]

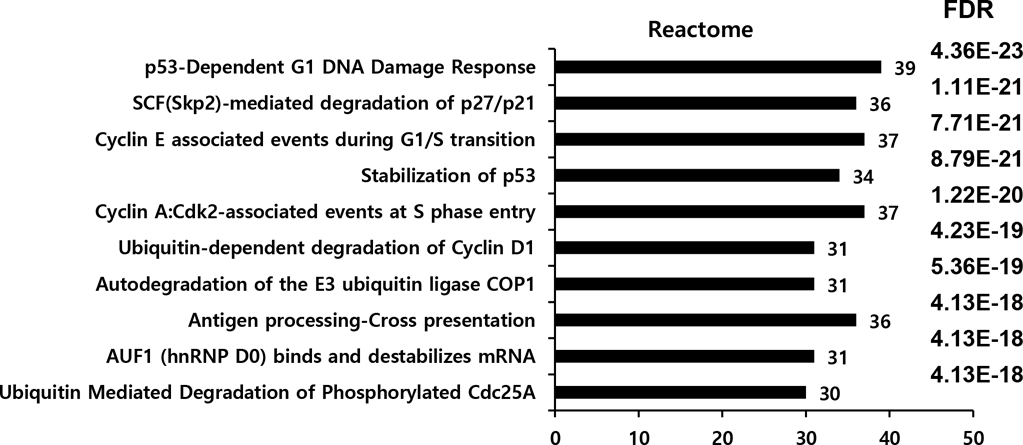

Supplement: Supplementary file 6 — Supplementary Figure 6 [file 41419_2020_2552_MOESM6_ESM.tif]

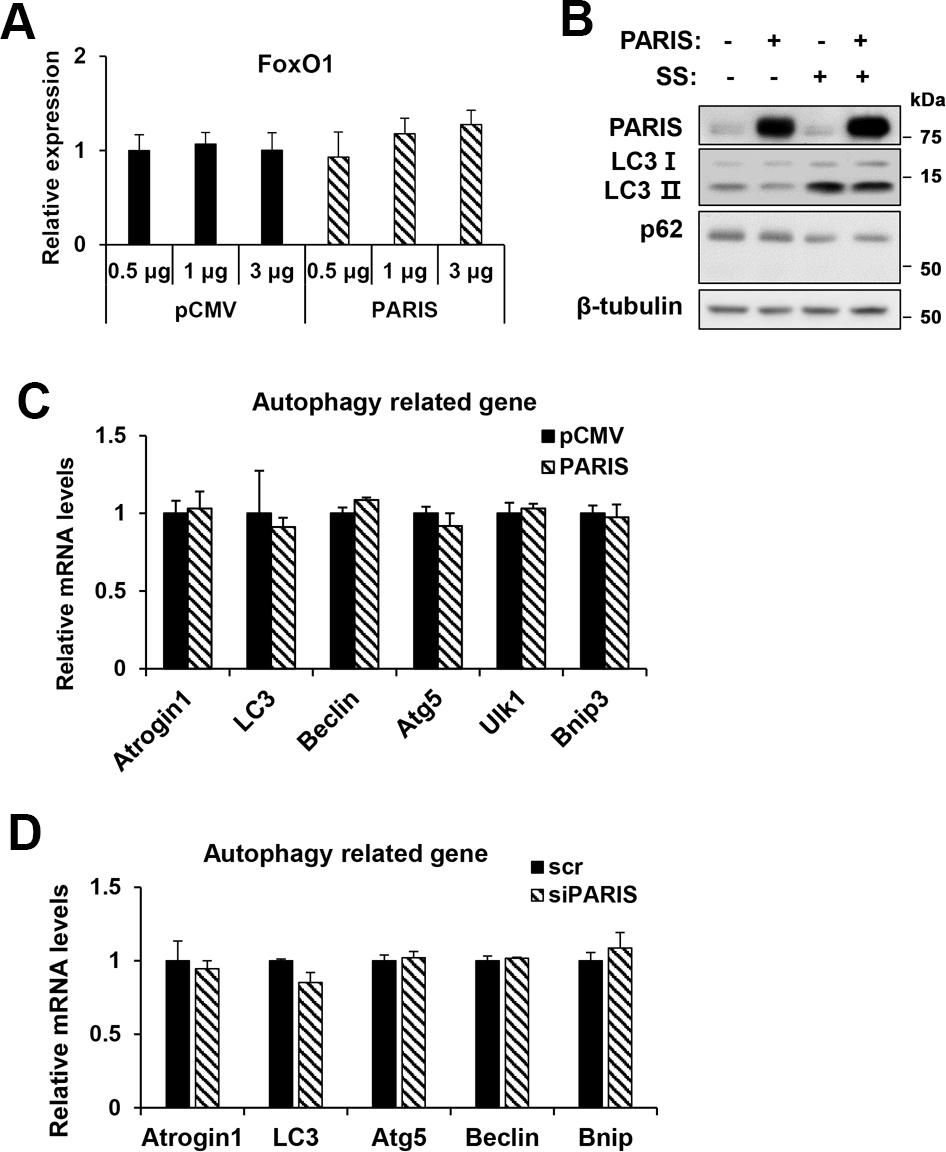

Supplement: Supplementary file 7 — Supplementary Figure 7 [file 41419_2020_2552_MOESM7_ESM.tif]

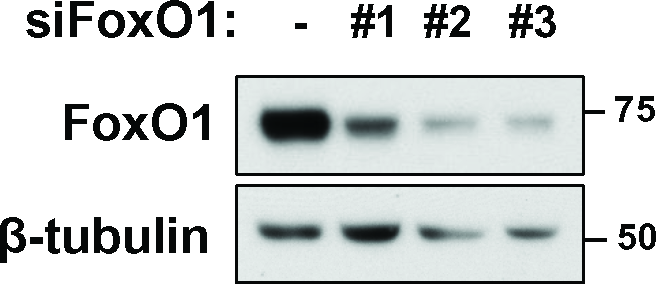

Supplement: Supplementary file 8 — Supplementary Figure 8 [file 41419_2020_2552_MOESM8_ESM.tif]

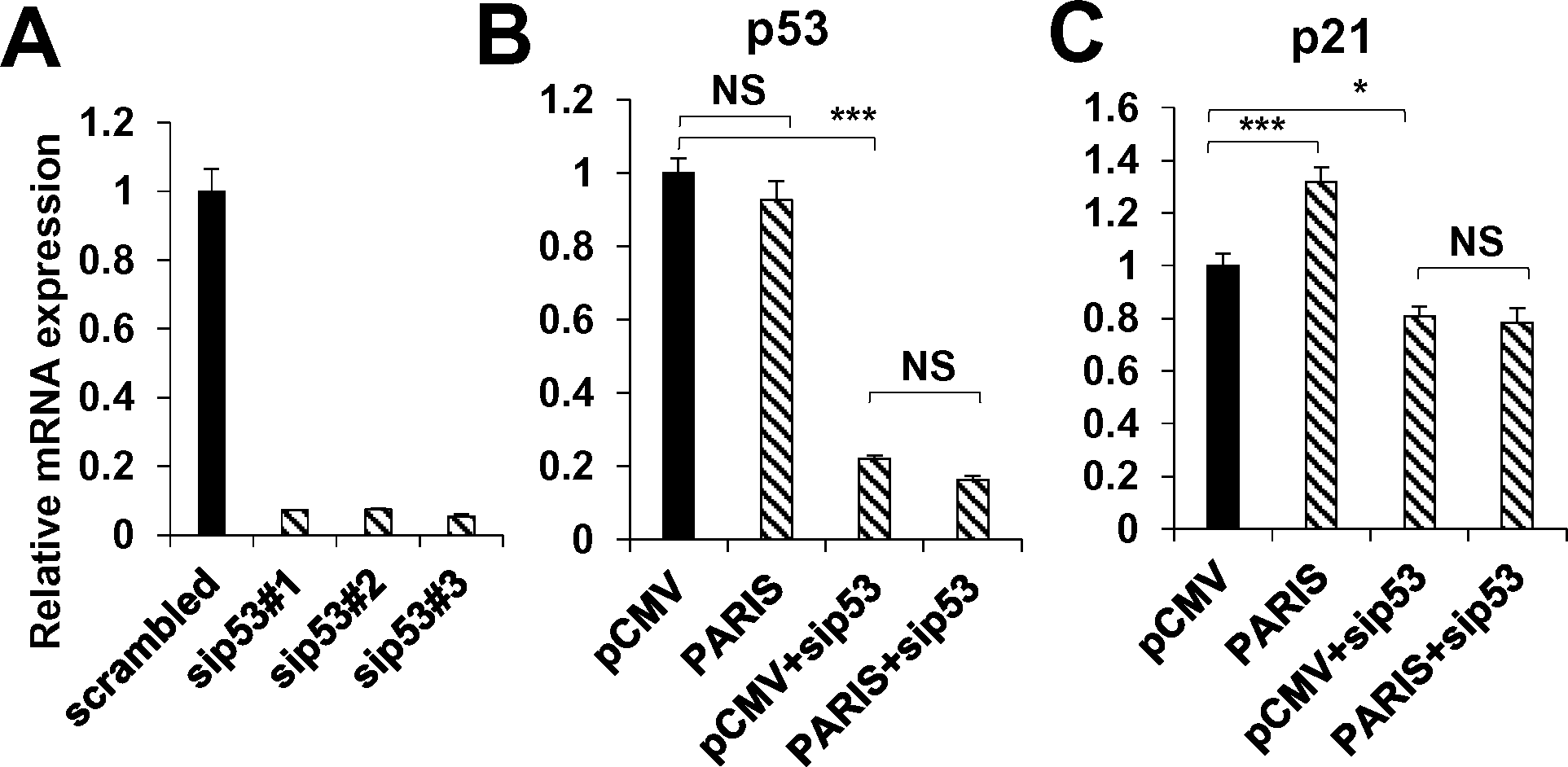

Supplement: Supplementary file 9 — Supplementary Figure 9 [file 41419_2020_2552_MOESM9_ESM.tif]
