# Supplementary material for: ZNF746/PARIS overexpression induces cellular senescence through FoxO1/p21 axis activation in myoblasts
Source: Cell Death Dis. 2020 May 12;11(5):359. doi: 10.1038/s41419-020-2552-7 (PMC7217926; doi:10.1038/s41419-020-2552-7)
Supplement: Supplementary file 10 — Table [file 41419_2020_2552_MOESM10_ESM.docx]

**Table**

**ZNF746/PARIS overexpression induces cellular senescence through FoxO1/p21 axis activation in myoblasts**

Ju-Hyeon Bae, Hyeon-Ju Jeong, Hyebeen Kim, Yong-Eun Leem, Dongryul Ryu, Sang Chul Park, Yun-Il Lee, Sung Chun Cho, Jong-Sun Kang

**Supplementary Table 1. siRNA sequence**

| **Genes** |  | **Sequence** |
| --- | --- | --- |
| **Scrambled siRNA** | Sense | UUCUCCGAACGUGUCACGUTT |
|  | anti-Sense | ACGUGACACGUUCGGAGAATT |
| **siPARIS #1** | Sense | CGUAUAGGAUCUUAAGUAAUU |
|  | anti-Sense | UUACUUAAGAUCCUAUACGUU |
| **siPARIS #2** | Sense | CCGAUUUCUCCAUGGACAAUGUU |
|  | anti-Sense | CAUUGUCCAUGGAGAAAUCGGUU |
| **siFoxO1 #1** | Sense | CCCAGUCUGUCUGAAAUCATT |
|  | anti-Sense | UGCUUUCAGACAGACUGGGTT |
| **siFoxO1 #2** | Sense | GCAACGAUGACUUUGAUAATT |
|  | anti-Sense | UUAUCAAAGUCAUCGUUGCTT |
| **siFoxO1 #3** | Sense | GAGGAUUGAACCAGUAUAATT |
|  | anti-Sense | UUAUACUGGUUCAAUCCUCTT |
| **sip53 #1** | Sense | GGACAGCCAAGUCUGUUAUTT |
|  | anti-Sense | AUAACAGACUUGGCUGUCCTT |
| **sip53 #2** | Sense | GACCUAUCCUUACCAUCAUTT |
|  | anti-Sense | AUGAUGGUAAGGAUAGGUCTT |
| **sip53 #3** | Sense | CCACUUGAUGGAGAGUAUUTT |
|  | anti-Sense | AAUACUCUCCAUCAAGUGGTT |

**Supplementary Table 2. Primer sequence**

| **Genes** | |  | **Sequence** |
| --- | --- | --- | --- |
| **PARIS** | | Forward | GTTGGAATGGACACCAGAGGT |
|  |  | Reverse | GGTTCCTGTGGACCCAAGTC |
| **PGC-1α** | | Forward | ATGTGTCGCCTTCTTGCTCT |
|  |  | Reverse | CGGTGTCTGTAGTGGCTTGA |
| **Sod1** | | Forward | CCAGTGCAGGACCTCATTTT |
|  |  | Reverse | CACCTTTGCCCAAGTCATCT |
| **Sod2** | | Forward | TTAACGCGCAGATCATGCA |
|  |  | Reverse | GGTGGCGTTGAGATTGTTCA |
| **Gpx1** | | Forward | GAAGAACTTGGGCCATTTGG |
|  |  | Reverse | TCTCGCCTGGCTCCTGTTT |
| **p21** | | Forward | AACATCTCAGGGCCGAAA |
|  |  | Reverse | TGCGCTTGGAGTGATAGAAA |
| **p53** | | Forward | GGAAATTTGTATCCCGAGTATCTG |
|  |  | Reverse | GTCTTCCAGTGTGATGATGGTAA |
| **FoxO1** | | Forward | CGTGCTTACAGCCTTCTA |
|  |  | Reverse | ACCTCCATCGTGACAAAA |
| **Il-6** | | Forward | GGTGACAACCACGGCCTTCCC |
|  |  | Reverse | AAGCCTCCGACTTGTGAAGTGGT |
| **Il-1α** | | Forward | GGAGAAGACCAGCCCGTGTTGCT |
|  |  | Reverse | CCGTGCCAGGTGCACCCGACTT |
| **Igfbp5** | | Forward | CTGGTGCCAAGGTGTTCTTGA |
|  |  | Reverse | CTCCAGAGTGATCCCTTTTTA CC |
| **Igfbp7** | | Forward | CTGGTGCCAAGGTGTTCTTGA |
|  |  | Reverse | CTCCAGAGTGATCCCTTTTTA CC |
| **Cxcl1** | | Forward | TGAGCTGCGCTGTCAGTGCCT |
|  |  | Reverse | AGAAGCCAGCGTTCACCAGA |
| **Cxcl10** | | Forward | CCACGTGTTGAGATCATTGCC |
|  |  | Reverse | GAGGCTCTCTGCTGTCCATC |
| **Rantes** | | Forward | CAGAGGAAAGAGAGAAAGTCC |
|  |  | Reverse | CACACGGTGACAGTGCTGG |
| **Tnf-α** | | Forward | AGCCCCCAGTCTGTATCCTT |
|  |  | Reverse | CTCCCTTTGCAGAACTCAGG |
| **L32** | | Forward | GGCCTCTGGTGAAGCCCAAGATCG |
|  |  | Reverse | CCTCTGGGTTTCCGCCAGTTTCGC |
| **Primer sequence for Chip assay** | | | |
|  | **Promoters (nt)** | | **Sequence** |
| **R1** | 620-647 | | CCTCCAACCATGTTTCTGAGTATACATT |
|  | 863-888 | | ATTGTCTGTCTGTTTACTTTGGGAGA |
| **R2** | 1253–1276 | | AACTCACAGCTTCTCCAAAGCAGG |
|  | 1501-1524 | | CATGTATGAAGCCAGGAGTTGGAT |
| **R3** | 2446-2471 | | ATGGGCTTGTTTTGTTTTTGAGAGGG |
|  | 2765-2789 | | TCTGTTGGTACAGTGTTTGCCTAAC |
