# Supplementary material for: ZNF746/PARIS overexpression induces cellular senescence through FoxO1/p21 axis activation in myoblasts
Source: Cell Death Dis. 2020 May 12;11(5):359. doi: 10.1038/s41419-020-2552-7 (PMC7217926; doi:10.1038/s41419-020-2552-7)
Supplement: Supplementary file 11 — Related Manuscript File [file 41419_2020_2552_MOESM11_ESM.docx]

**Supplementary Information**

**ZNF746/PARIS overexpression induces cellular senescence through FoxO1/p21 axis activation in myoblasts**

Ju-Hyeon Bae, Hyeon-Ju Jeong, Hyebeen Kim, Yong-Eun Leem, Dongryeol Ryu, Sang Chul Park, Yun-Il Lee, Sung Chun Cho, Jong-Sun Kang

**Supplementary Figure 1. The relative expression level of PARIS during myogenesis.**

(**A**) Relative protein expression level of PARIS shown in Figure 1A. Each level was normalized to that of β-tubulin, (3 sets) (**B**) Immunoblot analysis for the level of PARIS in Scrambled (Scr)- or two different PARIS siRNA (siPARIS, #1 and #2)-expressing C2C12 cells. β-tubulin was used as a loading control. Significant difference were determined by ANOVA, (panel A) (****p* < 0.001).

**Supplementary Figure 2. PARIS has no effect on cell death in myoblasts.**

(**A**) Apoptosis in pCMV- or pCMV-PARIS-overexpressing C2C12 cells was determined by FACS analysis of annexin-V-FITC and propidium iodide (PI). The cells treated with 1mM H_2_O_2_ for 1 hour were used as a control. (**B**) Immunoblot analysis for the level of Caspase3 and cleaved Caspase3 in pCMV- or pCMV-PARIS-overexpressing C2C12 cells. β-tubulin was used as a loading control.

**Supplementary Figure 3. PARIS was positively associated with senescence in MEF.**

(**A**) Cell passaging (P) was performed with primary MEFs isolated from E13.5 embryo. At each passage, the equal numbers of the cells (3X10^5^ cells) were seeded and 2 days later, cells were counted (3 sets per group). (**B**) Representative images of SA-β-Gal staining in P2 and P7 MEF cells. Scale bar=100μm. (**C**) Quantification of SA-β-Gal positive cells (n=3 per group, 179~239 (P2) and 123~139 (P7) cells in field were counted, respectively) (**D**) qRT-PCR analysis for SASP genes (IL-6, Igfbp5, Igfbp7, Cxcl3, TNF-α, and p21) in P2 and P7 MEF cells. Each value was normalized to an endogenous control L32 levels (3 sets per group). (**E**) The mRNA level of PARIS in MEFs during passaging shown in panel A. Each value was normalized to L32 levels (3 sets per group). (**F**) The protein expressions of PARIS and p21 in P2, P5 and P6 MEF cells. (**G**) The relative protein expression level of PARIS shown in panel F. Each level was normalized to that of β-tubulin, (3 sets per group). (**H**) Immunostaining for PARIS (green), Ki67 (red) in P7 MEF cells. Nuclei were visualized by DAPI staining (blue). Scale bar=50μm (**I**) Percentages of PARIS single positive (PARIS+/Ki67-), Ki67 single positive (PARIS-/Ki67+) and double positive (PARIS+/Ki67+) cells (n=5 per group, 16~28 (P2) and 17~46 (P7) cells in field were counted, respectively). Significant difference was determined by Student *t*-test (panel C and D) and ANOVA (panel G and I), (**p* < 0.05, ***p* < 0.01, ****p* < 0.001).

**Supplementary Figure 4. Increased expression of PARIS in doxorubicin-induced senescence of MEFs.**

MEFs were treated with doxorubicin (Dox, 50nM) for 4 days. (**A**) SA-β-gal staining of Dox treated MEFs and control. (**B**) Quantification of SA-β-gal-positive cells (n=3 per group, 276~346 (Control) and 29~39 (Dox) cells in field were counted, respectively). (**C**) Immunoblot analysis for PARIS and β-tubulin in MEFs. (**D**) qRT-PCR analysis for PARIS and p21 in MEFs, Each values was normalized to L32 (3 sets per group). Significant difference were determined by Student *t*-test (panel B and D), (***p* < 0.01, ****p* < 0.001).

**Supplementary Figure 5. Accumulation of lipofuscin in PARIS overexpressing C2C12 cells.**

Immunostaining of GL13 (green) and Flag (red) in C2C12 cells transfected with pCMV-PARIS-Flag plasmid. GL13 or PARIS were incubated with primary biotin- and primary Flag antibody, respectively. Nucleus was visualized by DAPI staining (blue). Scale bar=50μm.

**Supplementary Figure 6. Reactome analysis from RNA-seq data.**

Reactome analysis (1,121 genes (up=670, down=451)) for RNA-seq data shown in Figure 3A. Top 10 ranks are presented. Cut-off criteria are *p* < 0.05 and FDR *q*-value < 0.05. (n=3).

**Supplementary Figure 7. The overexpression of PARIS had no effect on autophagy.**

(**A**) The relative mRNA level of FoxO1 in C2C12 cells, which were transfected with 0.5, 1.0, or 3.0μg of pCMV or pCMV-PARIS plasmid. Each level was normalized to the level of L32 (3 sets per group). (**B**) Immunoblot analysis for PARIS, LC3 and p62 in PARIS-overexpressing 10T1/2 cells, which were induced to serum starvation (SS; 0.1% FBS containing media) for 24 hours or not at the confluence. β-tubulin serves as a loading control. (**C, D**) Real-time qRT-PCR analysis for expression of autophagy related genes in PARIS-overexpressing C2C12 cells (C) and PARIS-depleted C2C12 cells (D). Each value was normalized to L32 levels (3 sets per group).

**Supplementary Figure 8. The expression level of FoxO1 in different siRNA expressing C2C12.**

Immunoblot analysis for the level of FoxO1 in Scrambled (Scr)- or three different FoxO1 siRNA (siFoxO, #1, #2 and #3)-expressing C2C12 cells. β-tubulin was used as a loading control.

**Supplementary Figure 9. The expression of p21 is decreased by silencing p53 in PARIS overexpressing C2C12 cells.**

(**A**) C2C12 cells were transfected with Scrambled (Scr)- or different p53 siRNA (sip53, #1, #2 and #3). The level of p53 was assessed by qRT-PCR analysis. Each values was normalized to L32 an endogenous control (3 sets per group). (**B, C**) qRT-PCR analysis for p21 and p53 to examine the effect of p53 depletion with sip53-3 in control or PARIS-overexpressing C2C12 cells. Each value was normalized to L32 levels (3 sets per group). Significant difference was determined by ANOVA. (panel B and C), (NS = not significant, * *p* < 0.05, ***p* < 0.01, ****p* < 0.001).

**Supplementary Table 1. siRNA sequence**

| **Genes** |  | **Sequence** |
| --- | --- | --- |
| **Scrambled siRNA** | Sense | UUCUCCGAACGUGUCACGUTT |
|  | anti-Sense | ACGUGACACGUUCGGAGAATT |
| **siPARIS #1** | Sense | CGUAUAGGAUCUUAAGUAAUU |
|  | anti-Sense | UUACUUAAGAUCCUAUACGUU |
| **siPARIS #2** | Sense | CCGAUUUCUCCAUGGACAAUGUU |
|  | anti-Sense | CAUUGUCCAUGGAGAAAUCGGUU |
| **siFoxO1 #1** | Sense | CCCAGUCUGUCUGAAAUCATT |
|  | anti-Sense | UGCUUUCAGACAGACUGGGTT |
| **siFoxO1 #2** | Sense | GCAACGAUGACUUUGAUAATT |
|  | anti-Sense | UUAUCAAAGUCAUCGUUGCTT |
| **siFoxO1 #3** | Sense | GAGGAUUGAACCAGUAUAATT |
|  | anti-Sense | UUAUACUGGUUCAAUCCUCTT |
| **sip53 #1** | Sense | GGACAGCCAAGUCUGUUAUTT |
|  | anti-Sense | AUAACAGACUUGGCUGUCCTT |
| **sip53 #2** | Sense | GACCUAUCCUUACCAUCAUTT |
|  | anti-Sense | AUGAUGGUAAGGAUAGGUCTT |
| **sip53 #3** | Sense | CCACUUGAUGGAGAGUAUUTT |
|  | anti-Sense | AAUACUCUCCAUCAAGUGGTT |

**Supplementary Table 2. Primer sequence**

| **Genes** | |  | **Sequence** |
| --- | --- | --- | --- |
| **PARIS** | | Forward | GTTGGAATGGACACCAGAGGT |
|  |  | Reverse | GGTTCCTGTGGACCCAAGTC |
| **PGC-1α** | | Forward | ATGTGTCGCCTTCTTGCTCT |
|  |  | Reverse | CGGTGTCTGTAGTGGCTTGA |
| **Sod1** | | Forward | CCAGTGCAGGACCTCATTTT |
|  |  | Reverse | CACCTTTGCCCAAGTCATCT |
| **Sod2** | | Forward | TTAACGCGCAGATCATGCA |
|  |  | Reverse | GGTGGCGTTGAGATTGTTCA |
| **Gpx1** | | Forward | GAAGAACTTGGGCCATTTGG |
|  |  | Reverse | TCTCGCCTGGCTCCTGTTT |
| **p21** | | Forward | AACATCTCAGGGCCGAAA |
|  |  | Reverse | TGCGCTTGGAGTGATAGAAA |
| **p53** | | Forward | GGAAATTTGTATCCCGAGTATCTG |
|  |  | Reverse | GTCTTCCAGTGTGATGATGGTAA |
| **FoxO1** | | Forward | CGTGCTTACAGCCTTCTA |
|  |  | Reverse | ACCTCCATCGTGACAAAA |
| **Il-6** | | Forward | GGTGACAACCACGGCCTTCCC |
|  |  | Reverse | AAGCCTCCGACTTGTGAAGTGGT |
| **Il-1α** | | Forward | GGAGAAGACCAGCCCGTGTTGCT |
|  |  | Reverse | CCGTGCCAGGTGCACCCGACTT |
| **Igfbp5** | | Forward | CTGGTGCCAAGGTGTTCTTGA |
|  |  | Reverse | CTCCAGAGTGATCCCTTTTTA CC |
| **Igfbp7** | | Forward | CTGGTGCCAAGGTGTTCTTGA |
|  |  | Reverse | CTCCAGAGTGATCCCTTTTTA CC |
| **Cxcl1** | | Forward | TGAGCTGCGCTGTCAGTGCCT |
|  |  | Reverse | AGAAGCCAGCGTTCACCAGA |
| **Cxcl10** | | Forward | CCACGTGTTGAGATCATTGCC |
|  |  | Reverse | GAGGCTCTCTGCTGTCCATC |
| **Rantes** | | Forward | CAGAGGAAAGAGAGAAAGTCC |
|  |  | Reverse | CACACGGTGACAGTGCTGG |
| **Tnf-α** | | Forward | AGCCCCCAGTCTGTATCCTT |
|  |  | Reverse | CTCCCTTTGCAGAACTCAGG |
| **L32** | | Forward | GGCCTCTGGTGAAGCCCAAGATCG |
|  |  | Reverse | CCTCTGGGTTTCCGCCAGTTTCGC |
| **Primer sequence for Chip assay** | | | |
|  | **Promoters (nt)** | | **Sequence** |
| **R1** | 620-647 | | CCTCCAACCATGTTTCTGAGTATACATT |
|  | 863-888 | | ATTGTCTGTCTGTTTACTTTGGGAGA |
| **R2** | 1253–1276 | | AACTCACAGCTTCTCCAAAGCAGG |
|  | 1501-1524 | | CATGTATGAAGCCAGGAGTTGGAT |
| **R3** | 2446-2471 | | ATGGGCTTGTTTTGTTTTTGAGAGGG |
|  | 2765-2789 | | TCTGTTGGTACAGTGTTTGCCTAAC |
